# A hybrid and scalable brain-inspired robotic platform

**DOI:** 10.1038/s41598-020-73366-9

**Published:** 2020-10-23

**Authors:** Zhe Zou, Rong Zhao, Yujie Wu, Zheyu Yang, Lei Tian, Shuang Wu, Guanrui Wang, Yongchao Yu, Qi Zhao, Mingwang Chen, Jing Pei, Feng Chen, Youhui Zhang, Sen Song, Mingguo Zhao, Luping Shi

**Affiliations:** 1 Center for Brain-Inspired Computing Research (CBICR), Beijing Innovation Center for Future Chip, Optical Memory National Engineering Research Center, and Department of Precision Instrument, Tsinghua University, Beijing, 100084 China; 2 Department of Automation, Tsinghua University, Beijing, 100084 China; 3 Department of Computer Science and Technology, Tsinghua University, Beijing, 100084 China; 4 Department of Biomedical Engineering, Tsinghua University, Beijing, 100084 China

**Keywords:** Electrical and electronic engineering, Computer science, Energy science and technology, Mathematics and computing

## Abstract

Recent years have witnessed tremendous progress of intelligent robots brought about by mimicking human intelligence. However, current robots are still far from being able to handle multiple tasks in a dynamic environment as efficiently as humans. To cope with complexity and variability, further progress toward scalability and adaptability are essential for intelligent robots. Here, we report a brain-inspired robotic platform implemented by an unmanned bicycle that exhibits scalability of network scale, quantity and diversity to handle the changing needs of different scenarios. The platform adopts rich coding schemes and a trainable and scalable neural state machine, enabling flexible cooperation of hybrid networks. In addition, an embedded system is developed using a cross-paradigm neuromorphic chip to facilitate the implementation of diverse neural networks in spike or non-spike form. The platform achieved various real-time tasks concurrently in different real-world scenarios, providing a new pathway to enhance robots’ intelligence.

## Introduction

Humans have long aspired to develop an improved ability to handle multiple complex tasks in dynamic environments. Robots represent a physical manifestation of intelligence, particularly when placed in dynamic complex environments to make decisions and predictions. Although the operating principles of the human brain remain largely unknown, neuroscientific discoveries provide clues for designing intelligent robotic systems. Brain-inspired research has thus attracted widespread interest as a promising pathway for developing highly intelligent robotic platforms.

Significant breakthroughs have been made in brain-inspired computing paradigms and hardware over the past decade^1^. Inspired by the human brain’s hierarchical topologies and parallel-processing networks, various artificial neural networks (ANNs), particularly deep neural networks, have achieved unprecedented success in numerous machine learning tasks^2^. For example, convolutional neural networks (CNNs) have surpassed human-level performance in image recognition and classification^3,4^. Inspired by the spike patterns of human brain activity, spiking neural networks (SNNs) exhibit high bio-fidelity, rich coding with spatiotemporal information, and event-driven peculiarity, emerging as a prominent neural computing paradigm in processing dynamic sequential information with high energy efficiency^5,6^. Meanwhile, there is currently a trend toward integrating deep learning and neuroscience, providing a highly promising pathway to develop artificial general intelligence (AGI)^7,8^.

In parallel, rapid evolution in neural computing paradigms is also producing a proliferation of new types of computing hardware to accelerate computing. Distinct spike and non-spike computing paradigms have led to two developmental directions of computing hardware. Neural network accelerators are designed for optimizing operations in ANNs, such as ShiDianNao^9^, EIE^10^, and TPU^11^, which typically leverage parallel processing elements and efficient compression or data reuse. In contrast, neuromorphic chips support rich spatiotemporal bio-functionality, including Neurogrid^12^, TrueNorth^13^, SpiNNaker^14^, and Loihi^15^, providing high energy efficiency and event-driven representations. Some novel hybrid chip architectures have emerged, and Tianjic is currently the forerunner^16,17^.

Continued progresses in brain-inspired computing algorithms and hardware have resulted in substantial advancements in intelligent robots^18,19^. The intersection of robotics and neuroscience are endowing robots with intelligent perception, flexible movement and natural interactions with environments^20^. Some real-world applications have been demonstrated, including humanoid platforms^21^, robotic arms^22^, medical robots^23^, robot navigation^24^, and automated driving^25,26^ . These achievements have provided strategic opportunities to advance the design of intelligent robots. Most of these platforms, however, have been designed to be task-specific in simplified scenarios and have limited ability to perform multiple tasks simultaneously. Thus, a robotic platform with the capability to efficiently handle multiple complex tasks in a dynamic environment would be a valuable development.

To promote robotic research by mimicking human intelligence, in the current study we developed a hybrid and scalable intelligent robotic platform based on an unmanned bicycle with primary modules including visual, auditory, motion and decision-making, which can deal with multimodal tasks simultaneously. Development of the platform involved three major challenges. First, because multimodal data-flows are constantly changeable and involve various information channels in the time and space, it is difficult to gather and handle different types of information from external environments. Second, because the integration of individual modules requires a high-level planner, determining how to dispatch them to accomplish comprehensive system-level behaviors is a challenge. Third, because evolution and continuous learning are important features of the human brain, intelligent robots require scalability for network scale, quantity and diversity. However, it is difficult for a computing system to achieve this scalability due to hardware restrictions.

**Figure 1:**
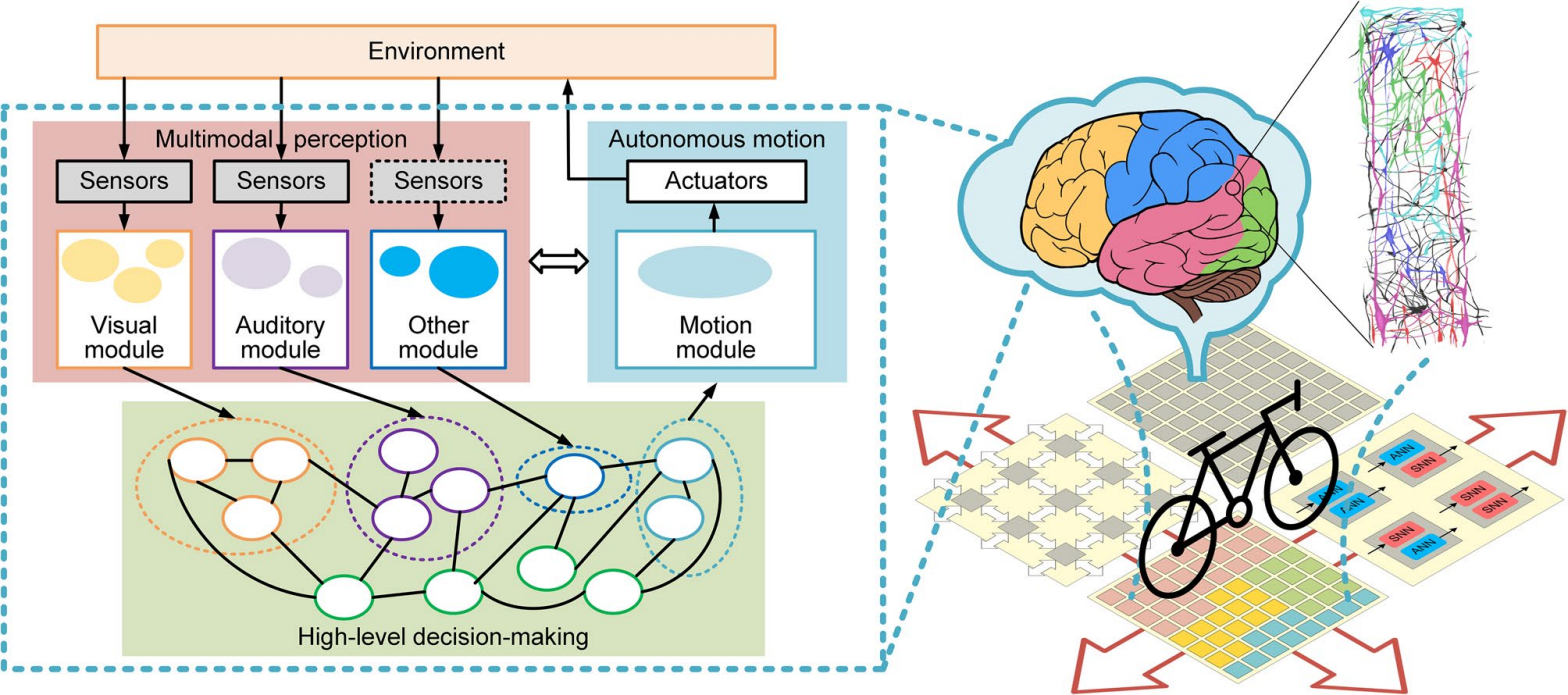
Intelligent architecture of the hybrid and scalable brain-inspired robotic platform. Software: Microsoft Visio 2019 MSO (16.0.10730.20102) 64-bit https://www.microsoft.com/en-us/microsoft-365/visio/flowchart-software**.**Adobe Photoshop version: 2015.0.0 20,150,529.r.88 2015/05/29:23:59:59 CL 1,024,429 × 64 https://www.adobe.com/products/photoshop.html.

To overcome the abovementioned challenges, we proposed three design principles to develop the robot platform, inspired by the human brain (Fig. 1). First, inspired by the functional specialization of the cerebral cortex^27^ and the rich coding schemes of biological neurons (rate, temporal, and population coding)^28^, we developed a hybrid architecture that can implement flexible inter-network cooperation and integrate different coding schemes efficiently. In this way, we can leverage the distinctive advantages of spiking and non-spiking neural networks in terms of energy efficiency and performance accuracy. Second, to adapt to dynamic environments, we developed a high-level decision-making module based on a hybrid neural state machine (HNSM) to integrate different modules flexibly, providing the capability to oversee and schedule different information flows, as well the capacity to be extended for dealing with increasing tasks during the implementation process. Third, inspired by neocortical regions organized with cortical columns^29^, we developed a scalable computing system based on our cross-paradigm neuromorphic chip, Tianjic, and a customized tool chain for hardware and software co-design^16,17^. The system has the potential to underpin brain-inspired system evolution and growth, similar to that exhibited in the human brain^30^.

On the basis of these design principles, we developed a systematic solution for building a brain-inspired robotic platform. The system architecture consisted of full network-based modules to interact with the environment and a cross-paradigm neuromorphic chip to support seamless integration of different neural networks. A set of approaches were implemented to improve the system performance, such as seamless transform for blending rich coding schemes, and network-based state machines for module cooperation. We experimentally demonstrated that the unmanned bicycle accomplished various real-time tasks concurrently, including object detection, tracking, voice command recognition, riding over a speed bump, obstacle avoidance, balance control, and decision-making in complex dynamic environments. Collectively, the scalability in both algorithms and hardware in terms of network scale, quantity and diversity enables the system-level complexity and continuous evolution to cope with the complex and dynamic environment. Such a hybrid and scalable robotic platform could enhance the development of intelligent robots.

## Results

In the following section, we first present a system-level overview of the robot platform focused on two aspects: intelligent architecture and the hardware system. Next, we elucidate three key features, including hybrid network-based module, flexible module cooperation and scalable neuromorphic computing. Finally, we demonstrate several implementations of the unmanned bicycle to evaluate our robot platform in real-world scenarios.

### System overview

**Figure 2:**
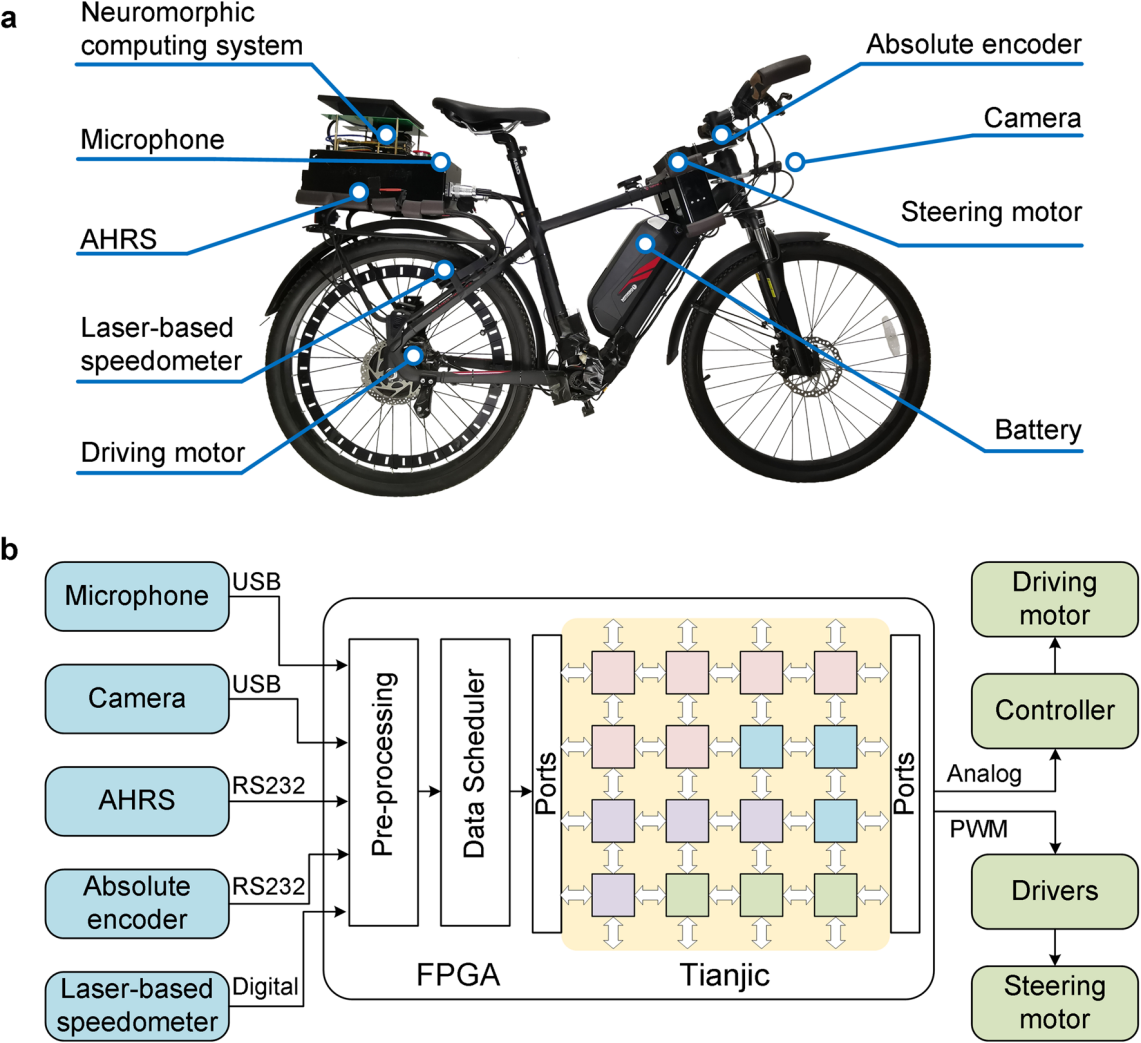
Hardware system overview of the robotic platform. (**a**) Mechanical structure with key sensors and electronic parts. (**b**) Hardware architecture with communications between the scalable neuromorphic computing system and the electronic devices. Software: Microsoft Visio 2019 MSO (16.0.10730.20102) 64-bit https://www.microsoft.com/en-us/microsoft-365/visio/flowchart-software.

- (1) Intelligent architecture. Our robotic platform adopted a hybrid-network modular design for perception, planning and execution. It consisted of visual, auditory, motion and decision-making modules as the primary configuration (see Fig. 1). Each module was built by a specialized neural network with spiking or non-spiking coding according to the spatiotemporal complexity of the data-flow, and can be improved iteratively with more training data or advanced learning rules. The HNSM-based decision module integrated multimodal information from the basic perception and provided instructions for motion. This hybrid and hierarchical organization together with the network-based decision module enables multi-network integration, flexible cooperation of multiple modules, rich coding schemes, and system scalability to deal with increasingly demanding tasks in a dynamic complex environment, providing the foundation of continuous evolution.
- (2) Hardware system. Our brain-inspired robotic platform was built on a modified electric bicycle^31^ equipped with a series of sensors and actuators to form a sensorimotor system with similarities to the human sensorimotor system (Fig. 2a). The platform collected multimodal information from its surroundings through a camera and a wireless microphone. A set of onboard sensors, including a laser-based speedometer, absolute encoder, attitude and heading reference system, worked together to determine the internal motion state of the platform. The movement control of the bicycle depended on a driving motor for speed change and a steering motor for balance-maintenance. All sensors and actuators were connected to a scalable neuromorphic computing system composed of an onboard Xilinx field programmable gate array (FPGA) and a Tianjic chip (Fig. 2b). The FPGA connected to sensors and actuators, and was dedicated to data acquisition, preprocessing and control instruction generation. Tianjic was used to implement hybrid architecture with diverse neural networks and rich coding schemes. The chip supported parallel processing of large networks or multiple networks by adopting a many-core architecture, reconfigurable building blocks and a streamlined data-flow with hybrid coding schemes.

It is worth to note that the system can be continuously improved by adding more modules to incorporate different types of sensors. For example, the visual module can fuse light detection and a ranging sensor, a dynamic vision sensor and other visual sensors. Moreover, the Tianjic chip is multi-chip extendable, which can upscale to support increased computing needs for multi-task implementation and increasingly complex environments.

### Hybrid network module

Efficient processing of multimodal data is challenging because multimodal information has different characteristics, such as the spatial nature of image information and the temporal correlation of voice messages. In addition, the arrival time of events, the amount of information, as well as the dimensionality of data, are all diverse.

**Figure 3:**
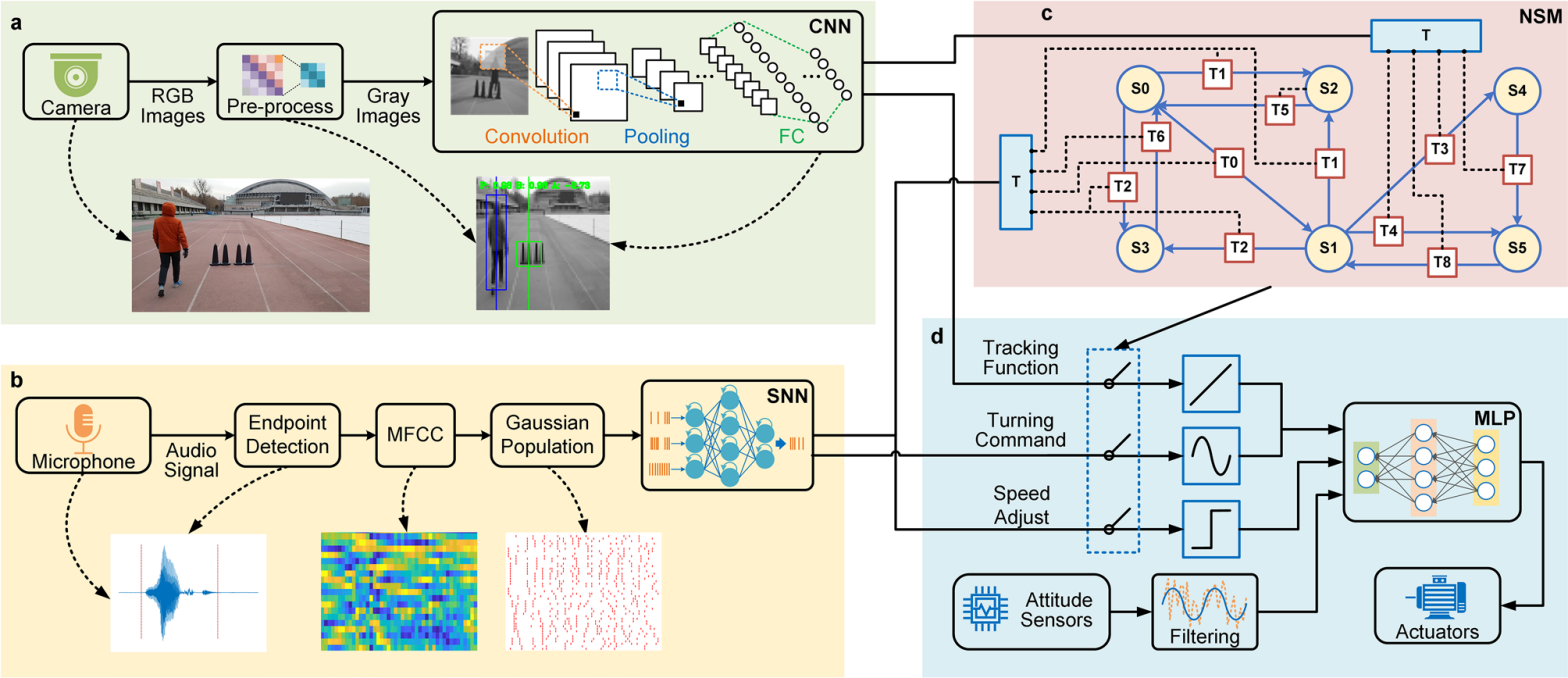
Networks structure and dataflow of the hybrid robotic platform. (**a**) Data flow diagram of CNN-based visual module and the illustration of CNN (input, structure and output). (**b**) SNN-based auditory module and the illustration of intermediate data. (**c**) HNSM-based decision module. (**d**) Sub-assemble parts and dataflow of motion module, including three function cores and an MLP. Software: Microsoft Visio 2019 MSO(16.0.10730.20102) 64-bit https://www.microsoft.com/en-us/microsoft-365/visio/flowchart-software.

Our hybrid module-based system with different types of networks was compatible with rich coding schemes, which can overcome the above challenge and handle multimodal tasks. An illustration of the hybrid architecture and network structure is presented in Fig. 3. The system combined hierarchical topology and parallel network processing. To deal with spatiotemporal multimodal data, each module was designed according to the characteristics of input data-flow. In general, spiking coding can naturally extract temporal correlations, and is more suited to handle sequence problems; in contrast, non-spiking coding, such as CNNs and MLPs, are more suitable for tasks with high spatial complexity. Thus, we developed a hybrid architecture that included a CNN-based visual module, an SNN-based auditory module and an MLP-based motion module. The HNSM-based decision module monitors different states of the platform, schedule diverse neural networks and fuse hybrid information flow between different modules.

The data-flow of each module as well as the communication between modules are also illustrated in Fig. 3. For the whole system, multimodal information from the external environment was captured through different sensors (camera, microphone), and sent to the corresponding module (visual, auditory) along different data-flows. High-level semantic information was then fed into the decision-making module to schedule various functional states. Finally, the motion module integrated internal and external signals to control the movement of the bicycle platform. Specially, these data-flows were a mixture of spiking and non-spiking coding. To seamlessly integrate hybrid coding schemes, we used various methods for signal transformation, which will be described later.

For each module, we opened independent data paths, which were distinct in information representation, frequency and throughput. In the visual module, each frame of video was resized to a 70 × 70 gray image and fed into a CNN as multi-bit values, enabling rich environmental spatial information to be maintained with limited computing resources (Fig. 3a). In contrast, the raw audio stream was transformed into binary spike trains in the auditory module. After end-point detection^32,33^, the key frequency features were obtained by taking the Mel Frequency Cepstral Coefficient (MFCC)^34^. A Gaussian population^35^ was used to encode each MFCC feature into spike trains as input to a three-layer fully-connected SNN (Fig. 3b). For motion control, sequential signals were first generated by functional cores to merge with the steering commands from other modules as the comprehensive target angle. Data from all sensors were then integrated via the MLP network for angle control (Fig. 3d). The designs and training of each network-based module are described in the Methods.

### Flexible module cooperation

The integration of diverse perception information and the cooperation of different functional modules is critical for the intelligent robotic platform. Currently, finite-state machines are commonly adopted in building a decision-making module. But they rely on predefined states and rules, lacking flexibility and scalability in networks, states and learning transfer rules to handle dynamic information with different time scales, metric levels, and spatiotemporal features.

Here, we designed a hybrid network-based state machine, HNSM, for high-level decision-making, which can not only fuse hybrid data-flows of spike and non-spike signals, but also be trained to cope with different complexity situations. An illustration of the structure and data-flows of the HNSM-based decision module is shown in Fig. 3c. The state machine transferred from one state to another depending on the external stimuli and internal state. For each state, relevant modules were activated in response to different situations. We used a conversion interface for signal fusion and a network-based model for learning rules so that the HNSM exhibited various properties that supported the hybrid and scalable platform.

To merge hybrid signals with different coding schemes, the input and output interfaces to transform different signals were designed in a unified form. All signals within HNSM propagated in spikes. The non-spike external signals were turned into spike trains to be consistent with the internal signals. For example, the multidigit value of the visual module was turned to one or zero by comparing with a threshold, while the spiking data of the auditory module remained intact. Thus, the HNSM had an event-driven attribute in response to changes in the environment. Furthermore, using a spiking information flow, the model could be reactive to multichannel signals with different frequencies. For example, when the operator gave a voice instruction or obstacles suddenly appeared, the HNSM received an external trigger and immediately changed to the response state. In addition, the accumulation mechanism of spikes was able to reduce interference. During obstacle detection, instead of single images, continuous signals are needed to determine whether obstacle recognition is stable, and the avoidance instruction is then generated.

**Figure 4:**
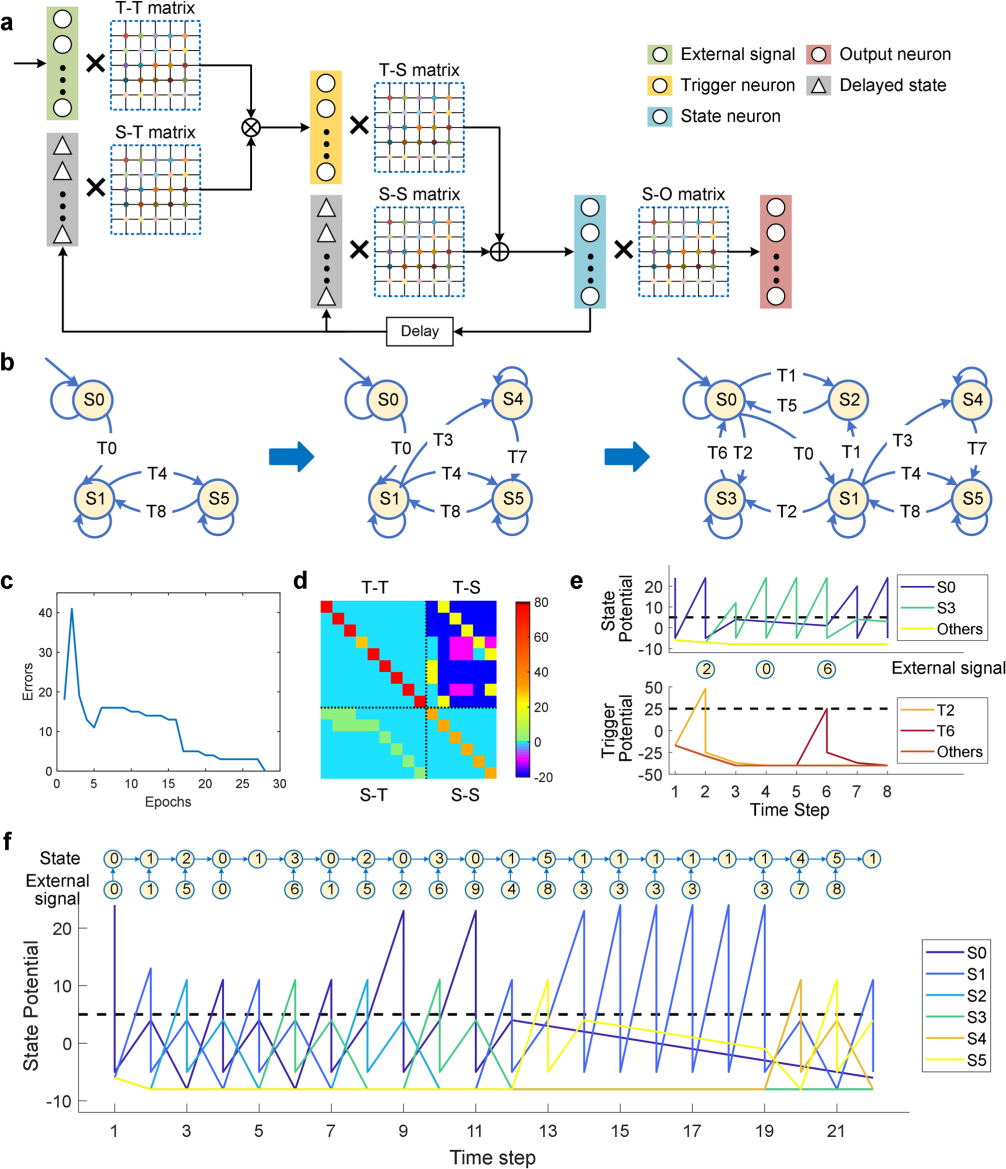
The development of HNSM and its results. (**a**) Evolution of HNSM in different task complexity. (**b**) Illustration of HNSM, including data flow, neuron definition, relationship of matrices and the whole structure. (**c**) The variation curve of error transfer during training. (**d**) The weights of T-T, T-S, S–S, S-T matrices. (**e**) State-maintenance and disturbance rejection. (**f**) Showcase of a complex state transition sequence. Software: Microsoft Visio 2019 MSO (16.0.10730.20102) 64-bit. https://www.microsoft.com/en-us/microsoft-365/visio/flowchart-software MATLAB R2017a (9.2.0.538062) 64-bit (win64) https://ww2.mathworks.cn/products/matlab.html.

The network-based state machine enables scalability to handle tasks with increasing complexity through training and self-learning. Our HNSM consists of three types of neuron populations: trigger (T), state (S) and output (O), and five connection matrices (T-T, T-S, S–S, S-T, S–O) (Fig. 4a). As a network-based model, the weight of connection matrices could be trained using sequences of pre-set states and external stimuli. More details regarding the states, triggers and training rules are provided in the Methods. Using this method, when environmental changes and new states are added, the model was able to learn transition rules automatically from data. The new states were introduced in two ways: (i) the original module with a single network was extended to accomplish more tasks; (ii) the HSNM accommodates additional modules. We performed a series of experiments to evaluate the scalable platform, starting with basic tasks, and then gradually increasing the network complexity (Fig. 4b). The visual module was trained to recognize tracking objects at beginning. The network was then expanding to identify obstacles concurrently. Thus, a new state (S4) was supplemented for avoiding obstacles. To promote interactions with humans, an auditory module was added for voice recognition. In addition, new states (S2, S3) were defined so that the robot could turn and change speed according to a command. Owning to the scalable decision module, the bicycle platform was able to continuously develop.

Figure 4c plots the training error curve during the training process, where errors referred to the fault activities of neurons compared with the supervisor, and were accumulated during each epoch for model evaluation. For each epoch, the weights were updated in 100 iterations. The error gradually declined and became zero at the end of training (see Methods section for details). The final weights of four matrices are shown in Fig. 4d. Due to the one-to-one correspondence between external signals and trigger neurons, the T-T matrix was a diagonal matrix. In addition, when there was no trigger or a wrong external signal, the current state should be maintained. Hence, the state neurons would continue to fire by themselves and the S–S was displayed in a diagonal matrix too.

Neuron activities were represented by the membrane potentials of both trigger and state neurons under a series of external signals, which can indicate state maintenance and transitions (Fig. 4e and f). When the potential accumulated and exceeded the threshold (black dotted line), the corresponding neuron fired, activating a trigger signal or a state output respectively. As each state only responded to specific triggers and other signals were treated as illegal, disturbances were suppressed leading to well-maintained states (Fig. 4e). Here, we used the current state as a switch. By multiplying with the state, the trigger neurons could filter out the illegal interference signals. Meanwhile, the state neuron would continue to maintain the current state. To evaluate the perform of HSNM and cooperation of networks, we designed a complex task with a transition sequence containing complete states to activate all modules of our hybrid robotic platform (Fig. 4f). The task involved all transition rules in Fig. 4b and each trigger or transition was executed immediately without any delay in between. This is an extreme and challenging test, because usually some states are maintained for a period of time before state trigger or transition, which is easier to perform. The results show that the system has flexible and flawless state transitions and a strong robustness against noise. When abnormal external stimuli were received (e.g., the blank trigger in Fig. 4f), false trigger signals were automatically shielded to ensure correct state transition. Hence, the interference disturbance caused by uncertain external stimuli from the environment were avoided, leading to stable outputs.

### Scalable neuromorphic computing

**Figure 5:**
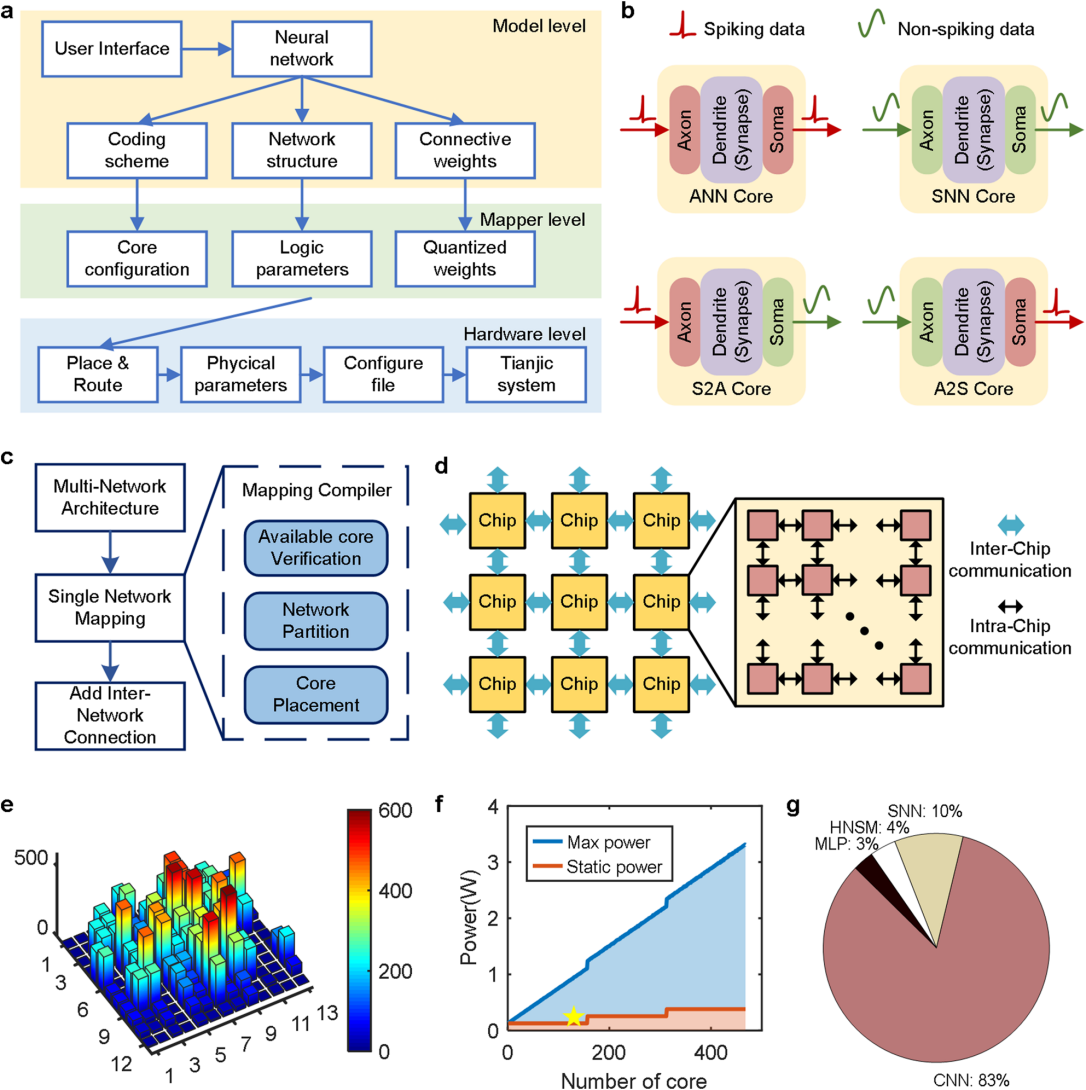
Scalable computing platform. (**a**) Three-level implementation architecture of Tianjic tool chain. (**b**) Four hybrid coding configurations for spiking and non-spiking dataflow. (**c**) Flow diagram of multi-network mapping strategy. (**d**) Illustration of hierarchy router structure. (**e**) Distribution of communication traffic in router for each core. (**f**) The power consumption for different core numbers. (**g**) Power consumption distribution of various networks. Software: Microsoft Visio 2019 MSO (16.0.10730.20102) 64-bit https://www.microsoft.com/en-us/microsoft-365/visio/flowchart-software MATLAB R2017a (9.2.0.538062) 64-bit (win64) https://ww2.mathworks.cn/products/matlab.html.

To cope with the complex environment and increasing difficulty of various tasks, intelligent systems should be able to scale up to accommodate more and/or larger networks. Utilizing the flexibility and the scalability provided by the HNSM at the software level, as well as an in-house developed tool chain that can map heterogeneous multiple networks, we built a flexible and scalable computing platform based on our cross-paradigm Tianjic chip. Furthermore, we developed a configuration process to transform, map, and execute hybrid models on the Tianjic chip so that the network-based models can be implemented on neuromorphic chip through software and hardware co-design. As illustrated in Fig. 5a, the implementation architecture had three levels: (1) model level: providing a user interface as well as abstracting the coding scheme, structure and weight from the original network; (2) mapper level: transforming the network to a hardware-friendly model and mapping it on logic cores; (3) hardware level: generating a hardware configuration file for running the model on the Tianjic system. Using this architecture, we were able to implement various networks on the Tianjic chip automatically.

The computing platform was able to support scalability in network scale, quantity and diversity through: (1) unified function cores with reconfigurable units for rich coding schemes and hybrid models; (2) automatic soft-hardware tools with flexible mapping strategies for multi-network implementation and incorporation; (3) arbitrary routing topology with a hierarchical 2D-mesh structure for large-scale communication and infinite extension.

First, for a hybrid network, the input and output of the core can be configured as an ANN or SNN model independently. Four types of combination, including ANN, SNN, ANN to SNN and SNN to ANN (A2A, S2S, A2S, S2A) can be generated for homogeneous or heterogeneous networks (Fig. 5b). In the bicycle experiment, most of the transformation cores (A2S and S2A) were located between two networks and acted as the interfaces.

Second, for multiple networks, they were divided into single networks and each network was deployed stepwise by the automatic tool chain without disturbance. The flow chart is shown in Fig. 5c. For a single network, the mapping complier transformed an original network model (usually a float model) to a hardware-friendly form. The parameters of the network were then allocated to multiple cores logically. Finally, the logical distribution was mapped to physical placement. At this stage, routing table configuration, physical layout, working mode and all configurations were determined by an automatic tool. After all networks were located, the tool added the connections between them. If the coding scheme of the network output was different from the next network, transformation cores were added and the target address of router was set as the transformation cores or the next network directly.

Third, for large-scale networks, we adopted some optimization mechanisms to ensure that multiple networks could run efficiently on the chip. These mechanisms included a point-to-point routing scheme and an adjacent multicast routing strategy^17^. The cores communicated with each other via a 2D-mesh method. Using this architecture, multiple cores can communicate with each other. In this way, large-scale parallel computing with any number and size of networks can be achieved by tiling cores and chips (Fig. 5d). We used a simulated annealing algorithm^36^ to reduce the number of routing packets transmitted on each transmission path and reduce the impact of communication transmission on chip computing. Figure 5e shows the distribution of communication traffic of routing passes cross cores. The input and output routing packets of each core changed depending on time-step. Here, we collected all packets over a period of time and displayed the maximum number of each core. All packets on the many-core architecture are almost evenly distributed, avoiding congestion in an individual core.

With the above three features, we were able to map the hybrid networks to Tianjic chip and achieve high-speed and low-power computing performance. The measurement and evaluation are described in the Methods. Table 1 lists the implementation performance of different networks in real-word environments. The clock was set to 300 MHz. All computations and communications were completed in 16.8 μs during each time phase, reflecting the minimum phase latency for guaranteeing accurate running performance. As shown in the table, the maximum event frequency did not exceed 200 Hz. Meanwhile, the running time of networks was less than the period of external signal, indicating that the system could handle all tasks in real-time. The many-core architecture enabled the real-time capability by tiling larger networks on more cores. The power consumptions of system with different scales are plotted in Fig. 5f. As core number increased, the system power consumption increased approximately linearly. The static power basically remained unchanged and only slightly increased when the core number exceeded the capacity of one chip.

**Table 1:** Implementation results of different networks.

Network	Structure	Core number	Event frequency (Hz)	Running time (ms)	Power (mW)
MLP	10–256-32–2	4	200	0.07	7.112
CNN	8C3-MP2-16C3-MP2-24C3-MP2-256–10	108	30	1.39	198.242
SNN	80–512-7	13	< 1	1.09	23.251
HNSM	6–9,9–9,9–6,6–6,6–6	5	200	0.07	8.845

For a real-world robotic system, the actual power consumption depends on practical requirements. These network-based modules handle different tasks. Hence, the scale of networks is different, which is the key influence factor on power consumption. In the bicycle experiment, 130 cores within one chip were used and the total dynamic power was 237.45 mW (marked as star in Fig. 5f), where the CNN for visual module occupied the largest part, about 83% (Fig. 5g). The computing system exhibited a high level of scalability for integrating the different scale, quantity and diversity of networks. Moreover, the system also has the potential for on-line multi-network reconstruction.

### System-level behaviors

**Figure 6:**
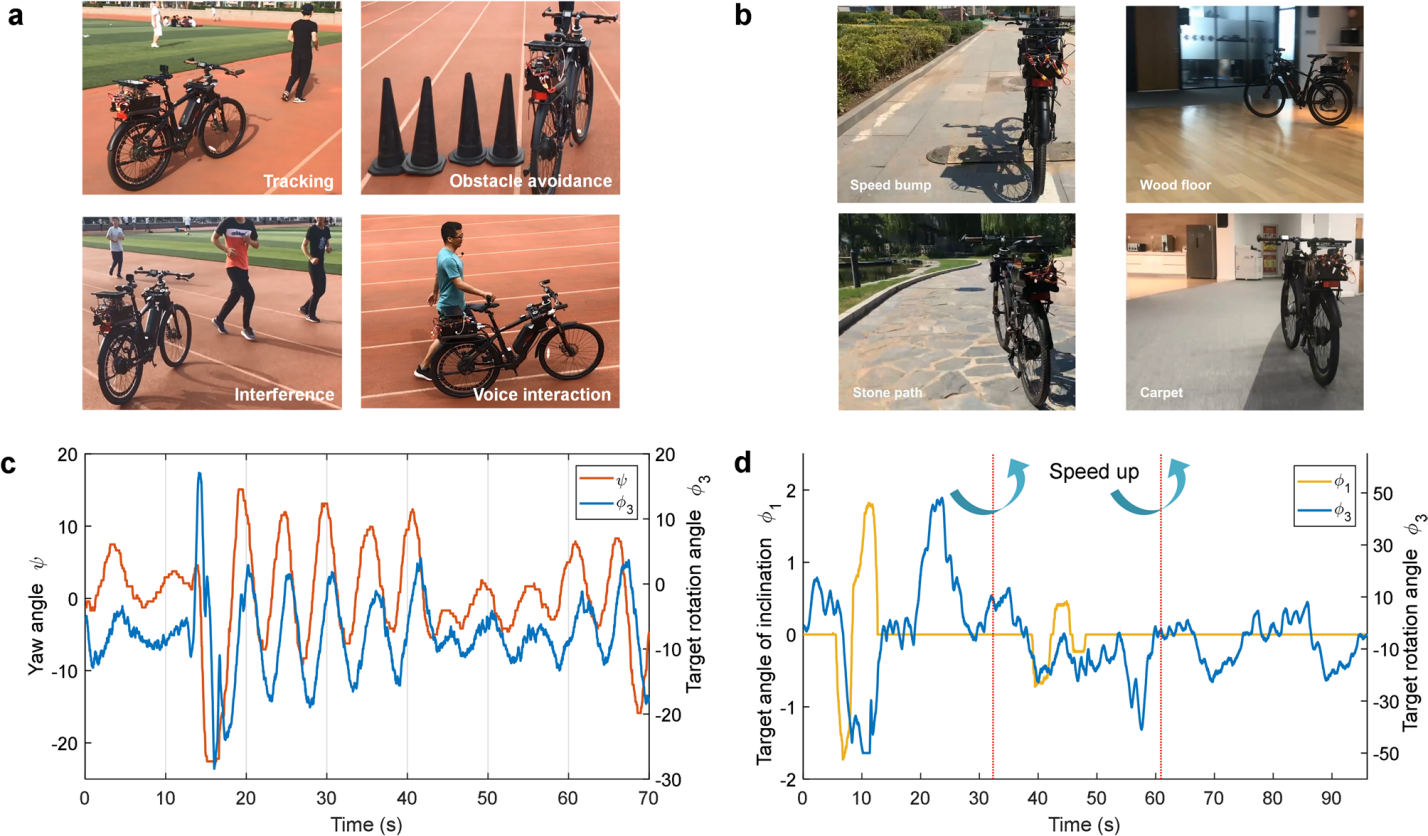
Illustration of system-level behaviors performance. (**a**) The actual dynamic and complex environment with multiple tasks. (**b**) Various real-world road conditions and corresponding specifications. (**c**) The plots of relative yaw angles of the bicycle, and the target rotation angles generated by control module. The left y axis (yaw angle) is converted from object position predicted by CNN, and the right y axis (target rotation angle) is generated by MLP for handlebar control. (**d**) The performance of MLP-based motion module on different speed level, commands and motion patterns. Software: Microsoft Visio 2019 MSO (16.0.10730.20102) 64-bit https://www.microsoft.com/en-us/microsoft-365/visio/flowchart-software MATLAB R2017a (9.2.0.538062) 64-bit (win64) https://ww2.mathworks.cn/products/matlab.html.

Robot operation in real world is challenging as it has to cope with many uncertainties. In this work, we evaluated our unmanned bicycle platform through a series of real-world tests, including different road conditions, various obstacles, and ambient noise. Figure 6a shows some examples of the real-world scenarios. The unmanned bicycle accomplished a comprehensive range of behaviors, including detecting and tracking a person, recognizing voice commands, and taking corresponding action.

When road conditions were varied, such as playground, grassland, stone path, and carpet, the bicycle was able to maintain balance easily and drive over speed bumps (Fig. 6b), which was benefitted from the robustness of MLP and the low latency of the system. The bicycle was able to follow a person who ran arbitrarily, and automatically avoided obstacles. The tracking and obstacle avoidance performance is presented in Fig. 6c. If another person come into view suddenly, the bicycle was able to keep tracking the original target. Figure 6d shows the results of different speed levels and multiple motion patterns. The target angle of inclination was generated from instructions. The bicycle executed a circular line, an S curve, and a straight line under different velocities, and accomplished stepless switching between different speed levels by only one network.

These tests demonstrate the effectiveness of our robotic platform in handling multiple tasks in dynamic variable environments. In summary, the hybrid intelligent architecture paves the way to enhance robots’ intelligence. Flexible multi-network cooperation based on HNSM and scalable software-hardware co-design system are the cornerstones for the hybrid and scalable brain-inspired robotic platform. In addition, it is worth to point out that this hybrid and scalable platform has the potential for iterative evolution by introducing more uncertainty and complexity, such as integrating more sensors and adding more functional modules for complex scenarios.

## Discussion

This work reports a hybrid and scalable brain-inspired robotic platform that achieves multiple complex tasks simultaneously, involving multimodal perception, high-level decision-making and autonomous motion. The fundamental design principles of this platform are inspired by the human brain, including a hybrid architecture for integrating different coding schemes, a high-level decision-making module for network cooperation and a scalable computing system for evolution. Based on this platform, an unmanned bicycle was developed, which accomplished various tasks concurrently, including object tracking, obstacle avoidance, voice command recognition, balance control, and decision-making in various real-world environments.

Our hybrid and scalable system can bring several unprecedented benefits and potentials. First, the hybrid architecture that integrates computer science and neuroscience-oriented approaches will benefit from the technological advances in these two fields, greatly promoting the development of brain-inspired robotic systems. Second, the excellent scalability in the platform, algorithms and computing capability will allow flexible integration of more sensors and functional modules to deal with complex scenarios. Third, the use of cross-paradigm neuromorphic computing system in the robot platform can not only support large-scale and diverse networks, but also promote the development of online learning.

In summary, the system can serve as a general platform for a wide range of robotics research from fundamental theory to applications, including perception, cognition, auto-control, language comprehension, decision-making, learning and adaptation. In addition, the hybrid and scalable platform can be developed iteratively and continuously improved. For example, complex problems with high spatiotemporal information can be generated by randomly introducing new variables into the environment, such as different road condition, noises, weather factors, multiple languages, and more people. By studying the adaptation to these environmental changes, we can investigate some key challenges of AGI, such as generalization, robustness, and autonomous learning, promoting the development of AGI.

## Methods

### CNN-based visual module

To accomplish object detection, we follow the idea of the YOLO^37^ and directly infers bounding box coordinates and class probabilities of objects from image pixels. The predicted objects are the tracking human and the obstacle. We recorded about one-hour video that contains the outdoor environment, humans and the obstacle as the training data. Due to the continuity of video streams, only 1 frame in every 10 frames is taken to ensure distinctions between two consecutive samples. So, the total dataset contains about 10 k frames. These frames are labelled by a pre-trained reliable Mask-RCNN^38^ model to get precise bounding boxes and probabilities. Then it is randomly horizontally flipped. The labels contain central coordinates (x, y) are augmented correspondingly. The brightness and contrast of the image is randomly adjusted with range [0.875, 1.125]and [0.5, 1.5], respectively as the training inputs.

Other than the model size, the bit-width of model parameters and intermediate data should also be addressed. Since Tianjic2 stores weights and propagate activations in INT8, our model should be quantized accordingly. Considering the chip’s hardware constraints, we use WAGE quantization method^39^ to restrict the bit-width of model parameters, where the bit-width of W, A, G, E is set to 8, 8, 32, and 32. Then we split the large convolution layer into smaller ones and distribute them into multi-cores.

### SNN-based auditory module

We establish a three-layer SNN with fully-connected structure. The network consists of the iterative leaky integrate-and-fire (LIF) neuron, which satisfies the neuronal membrane potential at time *t*, $o\left( t \right)$ is the neuronal firing state at time *t*, ${u}_{rest}$ following equation:

1 $$\left\{ {\begin{array}{l} {u\left( t \right) = u\left( {t_{{i - 1}} } \right)e^{{\frac{{t_{{i - 1}} - t}}{\tau }}} + I\left( t \right);} \hfill \\ {\left\{ {\begin{array}{l} {o\left( t \right) = 1,u\left( t \right) = u_{{rest}} \quad if\;u\left( t \right) \ge V_{{th}}; } \hfill \\ {o\left( t \right) = 0,\quad if\;u\left( t \right) < V_{{th}}, } \hfill \\ \end{array} } \right.} \hfill \\ \end{array} } \right.$$

where $u\left( t \right)$ is the neuronal membrane potential at time *t*, $o\left( t \right)$ is the neuronal firing state at time *t*, $u_{rest}$ is the resting potential, $V_{th}$ is the firing threshold and set to 0.5. $\tau$ is the membrane decay constant and set to 0.9, which makes it compatible with leakage adaption in our hardware soma unit. At the output, we use the one-hot coding, which means that each output neuron represents one instruction. And we introduce a background neuron which corresponds to the case where network does not issue an instruction.

Since the spike signals not only propagate along the layer-by-layer spatial domain, but also along the temporal domain via the well-known leaky-and-integrating mechanism, we adopt the emerging spatiotemporal backpropagation algorithm^40^ to train our model. This method converts the LIF model into an explicit expression, to be friendlier to back propagation, and compute the gradient information along the spatial dimension and temporal dimension for further employing the spatiotemporal dynamic of spiking neurons. Also, according to^35^, we use the rectangle function to approximate the non-differential points at spike firing times.

To evaluate the SNN performance, we take the similar mean square error function as loss function to measure the discrepancy between the averaged output results and the ground true command *Y* within a given time window *T*, and it yield

2 $$L = \left\| {Y - \frac{1}{T}\sum\nolimits_{t = 1}^{T} {o_{t}^{N} } } \right\|_{2}^{2},$$

where $o_{t}^{N}$ denotes the output of network in the last layer N at time *t*. We set the sampling window T to 30 and max epoch to 200. Because each instruction has a different number of frames, we used the stochastic gradient descent method to train model. Also, we adopt the Adam (adaptive moment estimation method to accelerate convergence and set the hyper-parameters $\beta_{1} ,\beta_{2} ,\lambda$ to 0.9, 0.999, $1{ - }10^{{{ - }8}}$. After quantized in INT8 and deployed on neuromorphic chip, the practical accuracy is 90.52%. Results show that the network can gain a high precision on both dataset and real environment.

### MLP model for motion control

The controller aggregates information of three motion sensors, one target angle and one speed coefficient, then outputs one target rotation angle to motor controllers. The descriptions of six signals are shown in in Supplementary Table 1. Inputs were first scaled according to their physical domain and then quantized to INT8, followed by the concatenation process. Finally, they were fed into the MLP at 50 kHz. The single output was sent to the motor controller to tune the steering for balance maintaining, and human tracking or obstacle avoidance. Besides, the voltage coefficient was directly sent to another motor (back wheel) controller to adjust the speed. The total response time for a single input is about 60us (not include the motor), which we find is a suitable frequency to control the bicycle.

The MLP receives all kinds of sensors signal, and none of any prior knowledge or dynamics of the bicycle are used. To bring in historical information, we recall signals from the latest T-time steps and concatenate them as the final input for MLP. The length of history recall can reflect the high order information in time dimension. The length of history recall is optimized according to both the testing error and the control result on actual bicycle. When recalling too much historical information (> 10 time steps), the handle bar seems to delay too long for turning and results in oscillation, even though its imitation loss (mean-square error) is lower, which indicates an overfitting problem of neural networks.

### Design and training of HNSM

In the bicycle experiment, the HNSM consists of 6 states (S0-S5) and 9 triggers (T0-T8). The definition of states and triggers are shown in Supplementary Table 2. The experiment starts from the initial state (S0), where the bicycle goes straight and waits for instructions. When a voice command is sent out and recognized, the auditory module generates a trigger signal and automatically switches the state according to the command. If the command is “speed up” or “slow down” (T1), the bicycle executes the corresponding command for speed change (S2) and then returns to S0 immediately by generating an internal trigger (T5). If the command is “left” or “right” (T2), it will be turning until a “straight” command (T6) is sent out. If the command is “follow me” (T0), it switches to the target tracking state (S1) so that the visual module is activated. The bicycle will then autonomously follow the person and take action in response to environmental interferences. For example, if an obstacle is detected, the bicycle will execute a ‘force turn’ instruction for obstacle avoiding (S4). During this period, all stimulations are blocked until the turn is completed. When the tracking object is out of view, the bicycle goes straight and seeks people (S5). The state will remain until tracking target is detected.

The HNSM is trained using supervised learning. The training data are collected from real-world experiment or generated from simulation environment. They are sequences of states, triggers and outputs, labeled as the ‘supervisory command’ (command includes state, trigger and output) at every time-step. We proposed a decoupling training method to train all matrices separately. For each matrix, the activities of relative neurons are variable and other neurons are set to the force command. For example, when training the S-T matrix, the output of S-T is compared with ‘supervisory trigger’ and the state is set to ‘supervisory state’ as the input. For each synapse in the axon-neuronal matrix, the weights are learned by an STDP-like rule:

3 $$\left\{ \begin{array}{l} W_{ij} = W_{ij} + \delta ,f(I_{i} )\& \overline{f} (O_{j} )\& f(S_{j} ); \hfill \\ W_{ij} = W_{ij} - \delta ,f(I_{i} )\& f(O_{j} )\& \overline{f} (S_{j} ), \hfill \\ \end{array} \right.$$

where $W_{ij}$ denotes the synapse weight of the i-th input and the j-th output, $I,O,S$ denotes input, output and supervisor respectively, $\delta$ denotes the adjustment parameter and set to 0.1. $f(\cdot)$ describes whether the neuron is fired, which set to 1 when the neuron fired and set to 0 if not. The training is required to be performed by several learning epochs and iteration in each epoch to get stable synapse weights. In the bicycle experiment, the maximum number of iterations set to 100. There is a one-to-one correspondence between the state and output, so the S–O matrix is an identity matrix and can be omitted.

### Characteristics of neuromorphic computing chip

The scalability of the Tianjic architecture derives from its modular many-core structure. When cores are tiled on the chip and connected by a network on chip, these small bipartite graphs are combined to form a larger neural network. Each chip consists of 156 reconfigurable unified cores. Each core has five building blocks: axon for hybrid activation and spike buffer, dendrite for shared integration with a local synapse memory, soma for nonlinear neural transformation, and router for inter-core connection. These building blocks can be flexibly reconfigured to support different NN modes, and the resources are greatly shared. The unified routing packet scheme transmitted between cores can be parsed as either a multi-value activation data in ANN mode or a binary spiking event in SNN mode. Tianjic chip was fabricated using a 28-nm high performance low power technology. It has a on chip memory with total number of roughly 22 KB static random-access memory (SRAM) in each core. The max power of one chip is around 1.1 W when clock is 300 MHz and voltage is 0.9 V, where consumption of single core is 5.56mW (SNN mode) and 6.27mW (ANN mode). The specification indexes show high performance of the Tianjic chip compared with existing neural network platforms, which is suitable to realize high-speed and low-power computing system.

### Evaluation of network hardware implementation

The computing system of bicycle is based on a single-chip PCB equipped with an Altera Cyclone 4 FPGA and communication interfaces like USB and SPI. We mapped the MLP, CNN, SNN and HNSM on Tianjic successively. To evaluate the performance of network implementation, we built a platform which provides input to the computing system and can test the power consumption. During the test, we first turned on different networks separately to test their consumption of processing single task. Then we measured the overall consumption of the network. According to requirements of bicycles in real-world, MLP and HNSM for control were producing 200 Hz control signals, CNN for visual were processing images at the speed of 30 fps, and the SNN for audio were recognizing voice commands received every second. In actual experiment, the system runs at 0.9 V, 300 M working conditions, and the total energy consumption is less than 0.4 W.

## Supplementary information

Supplementary information

## References

[1] Roy K, Jaiswal A, Panda P (2019). Towards spike-based machine intelligence with neuromorphic computing. Nature.

[2] LeCun Y, Bengio Y, Hinton G (2015). Deep learning. Nature.

[3] A. Krizhevsky, I. Sutskever, G. E. Hinton, in *Advances in Neural Information Processing Systems* (2012), pp. 1097–1105.

[4] K. He, X. Zhang, S. Ren, J. Sun, in *Proceedings of the IEEE Conference on Computer Vision and Pattern Recognition* (2016), pp. 770–778.

[5] Bichler O, Querlioz D, Thorpe SJ, Bourgoin J-P, Gamrat C (2012). Extraction of temporally correlated features from dynamic vision sensors with spike-timing-dependent plasticity. Neural Netw..

[6] Cao Y, Chen Y, Khosla D (2015). Spiking deep convolutional neural networks for energy-efficient object recognition. Int. J. Comput. Vis..

[7] Ullman S (2019). Using neuroscience to develop artificial intelligence. Science.

[8] Marblestone AH, Wayne G, Kording KP (2016). Toward an integration of deep learning and neuroscience. Front. Comput. Neurosci..

[9] Z. Du, R. Fasthuber, T. Chen, P. Ienne, L. Li, T. Luo, X. Feng, Y. Chen, O. Temam, in *2015 ACM/IEEE 42*nd* Annual International Symposium on Computer Architecture (ISCA), 13-17 June 2015, Portland, OR, USA*, pp. 92–104

[10] Han S, Liu X, Mao H, Pu J, Pedram A, Horowitz MA, Dally WJ (2016). EIE. SIGARCH Comput. Archit. News.

[11] Jouppi, N. P. *et al.* In-datacenter performance analysis of a tensor processing unit. In *Proceedings of the 44th Annual International Symposium on Computer Architecture* Vol. 17 1–12 (2017).

[12] Benjamin BV, Gao P, McQuinn E, Choudhary S, Chandrasekaran AR, Bussat J-M, Alvarez-Icaza R, Arthur JV, Merolla PA, Boahen K (2014). Neurogrid: a mixed-analog-digital multichip system for large-scale neural simulations. Proc. IEEE.

[13] Merolla PA, Arthur JV, Alvarez-Icaza R, Cassidy AS, Sawada J, Akopyan F, Jackson BL, Imam N, Guo C, Nakamura Y, Brezzo B, Vo I, Esser SK, Appuswamy R, Taba B, Amir A, Flickner MD, Risk WP, Manohar R, Modha DS (2014). A million spiking-neuron integrated circuit with a scalable communication network and interface. Science.

[14] Furber SB, Galluppi F, Temple S, Plana LA (2014). The SpiNNaker project. Proc. IEEE.

[15] Davies M, Srinivasa N, Lin T-H, Chinya G, Cao Y, Choday SH, Dimou G, Joshi P, Imam N, Jain S, Liao Y, Lin C-K, Lines A, Liu R, Mathaikutty D, McCoy S, Paul A, Tse J, Venkataramanan G, Weng Y-H, Wild A, Yang Y, Wang H (2018). Loihi: a neuromorphic manycore processor with on-chip learning. IEEE Micro..

[16] Pei J, Deng L, Song S, Zhao M, Zhang Y, Wu S, Wang G, Zou Z, Wu Z, He W, Chen F, Deng N, Wu S, Wang Y, Wu Y, Yang Z, Ma C, Li G, Han W, Li H, Wu H, Zhao R, Xie Y, Shi L (2019). Towards artificial general intelligence with hybrid Tianjic chip architecture. Nature.

[17] L. Deng, J. Pei, Z. Wu, X. Hu, Y. Ding, W. He, Y. Xie, L. Shi, G. Wang, G. Li, S. Li, L. Liang, M. Zhu, Y. Wu, Z. Yang, Z. Zou, Tianjic: a unified and scalable chip bridging spike-based and continuous neural computation. *IEEE J. Solid-State Circuits*, 1–19 (2020).

[18] Floreano D, Ijspeert AJ, Schaal S (2014). Robotics and neuroscience. Curr. Biol..

[19] Krichmar JL (2018). Neurorobotics—a thriving community and a promising pathway toward intelligent cognitive robots. Front. Neurorobot..

[20] S. Sanders, J. Oberst, Brain-inspired intelligent robotics: The intersection of robotics and neuroscience. *Sci. /AAAS*, 1–53 (2016).

[21] Tsagarakis NG, Metta G, Sandini G, Vernon D, Beira R, Becchi F, Righetti L, Santos-Victor J, Ijspeert AJ, Carrozza MC, Caldwell DG (2007). iCub: the design and realization of an open humanoid platform for cognitive and neuroscience research. Adv. Robot..

[22] N. Fazeli, M. Oller, J. Wu, Z. Wu, J. B. Tenenbaum, A. Rodriguez, See, feel, act: Hierarchical learning for complex manipulation skills with multisensory fusion. *Sci. Robot.* **4**, eaav3123 (2019). 10.1126/scirobotics.aav3123 33137764

[23] Chen AI, Balter ML, Maguire TJ, Yarmush ML (2020). Deep learning robotic guidance for autonomous vascular access. Nat. Mach. Intell..

[24] Milde MB, Blum H, Dietmüller A, Sumislawska D, Conradt J, Indiveri G, Sandamirskaya Y (2017). Obstacle avoidance and target acquisition for robot navigation using a mixed signal analog/digital neuromorphic processing system. Front. Neurorobot..

[25] K. D. Fischl, G. Tognetti, D. R. Mendat, G. Orchard, J. Rattray, C. Sapsanis, L. F. Campbell, L. Elphage, T. E. Niebur, A. Pasciaroni, V. E. Rennoll, H. Romney, S. Walker, P. O. Pouliquen, A. G. Andreou, in *2017 51*st* Annual Conference on Information Sciences and Systems (CISS), 22–24 March 2017*, pp. 1–6.

[26] N. A. Spielberg, M. Brown, N. R. Kapania, J. C. Kegelman, J. C. Gerdes, Neural network vehicle models for high-performance automated driving. *Sci. Robot.* **4** (2019). 10.1126/scirobotics.aaw1975 33137751

[27] H.-J. Park, K. Friston, Structural and functional brain networks: from connections to cognition. *Science* **342** (2013). 10.1126/science.1238411 24179229

[28] Kumar A, Rotter S, Aertsen A (2010). Spiking activity propagation in neuronal networks: reconciling different perspectives on neural coding. Nat. Rev. Neurosci..

[29] J. C. Horton, D. L. Adams, The cortical column: a structure without a function. *Philos. Trans. R. Soc.Lond. Ser. B, Biol. Sci.* **360**, 837–862 (2005). 10.1098/rstb.2005.1623 PMC1569491 15937015

[30] R. Glenn Northcutt, J. H. Kaas, The emergence and evolution of mammalian neocortex. *Trends Neurosci.* **18**, 373–379 (1995). 10.1016/0166-2236(95)93932-n 7482801

[31] J. He, M. Zhao, S. Stasinopoulos, in *2015 IEEE International Conference on Robotics and Biomimetics, December 6–9, 2015, Zhuhai, China*, pp. 428–433.

[32] Owens FJ (1993). Signal Processing of Speech.

[33] S. Jia-lin, H. Jeih-weih, & L. Lin-shan, Robust entropy-based endpoint detection for speech recognition in noisy environments (1998).

[34] Vergin R, O’Shaughnessy D, Farhat A (1999). Generalized mel frequency cepstral coefficients for large-vocabulary speaker-independent continuous-speech recognition. IEEE Trans. Speech Audio Process..

[35] Mathis A, Herz AVM, Stemmler MB (2012). Resolution of nested neuronal representations can be exponential in the number of neurons. Phys. Rev. Lett..

[36] van Laarhoven, Peter J. M., E. H. L. Aarts, in *Simulated Annealing: Theory and Applications*, 1987, Springer Netherlands, Dordrecht, pp. 7–15.

[37] J. Redmon, S. Divvala, R. Girshick, A. Farhadi, in *Proceedings of the IEEE Conference on Computer Vision and Pattern Recognition* (2016), pp. 779–788.

[38] K. He, G. Gkioxari, P. Dollár, R. Girshick, Mask R-CNN, in *Proceedings of the IEEE International Conference on Computer Vision* (2017), pp. 2980–2988.

[39] S. Wu, G. Li, F. Chen, L. Shi, Training and inference with integers in deep neural networks.in *International Conference on Learning Representations* (2018).

[40] Wu Y, Deng L, Li G, Zhu J, Shi L (2018). Spatio-Temporal Backpropagation for Training High-Performance Spiking Neural Networks. Front. Neurosci..

