# Supplementary material for: A hybrid and scalable brain-inspired robotic platform
Source: Sci Rep. 2020 Oct 23;10:18160. doi: 10.1038/s41598-020-73366-9 (PMC7584638; doi:10.1038/s41598-020-73366-9)
Supplement: Supplementary file 1 — Supplementary information [file 41598_2020_73366_MOESM1_ESM.pdf]

Supplementary Information for

A hybrid and scalable brain-inspired robotic platform

Zhe Zou[1], Rong Zhao[1], Yujie Wu[1], Zheyu Yang[1], Lei Tian[1], Shuang Wu[1], Guanrui Wang[1], Yongchao Yu[2], Qi Zhao[1], Mingwang Chen[1], Jing Pei[1], Feng Chen[2], Youhui Zhang[3], Sen Song[4], Mingguo Zhao[1,2]*, and Luping Shi[1]*

Affiliations

[1]Center for Brain-Inspired Computing Research (CBICR), Beijing Innovation Center for Future Chip, Optical Memory National Engineering Research Center, & Department of Precision Instrument, Tsinghua University, Beijing 100084, China

[2]Department of Automation, Tsinghua University, Beijing 100084, China

[3]Department of Computer Science and Technology, Tsinghua University, Beijing 100084, China

[4]Department of Biomedical Engineering, Tsinghua University, Beijing 100084, China

*Corresponding author. e-mail: mgzhao@tsinghua.edu.cn
lpshi@tsinghua.edu.cn

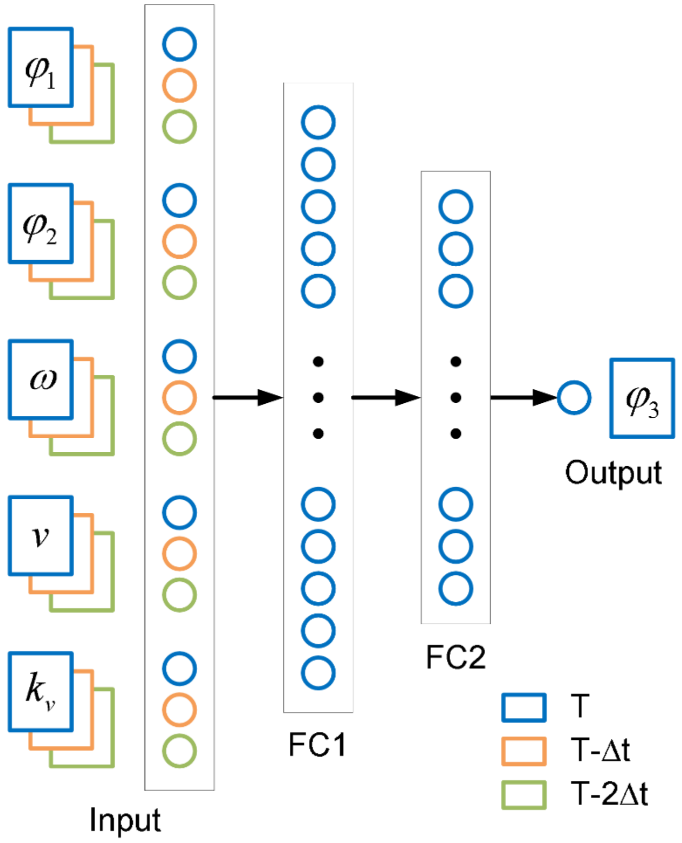

Supplementary Figure 1. The network structure of MLP. The inputs are the concatenation of signals from the latest T-k*Δt (time steps).The network consists of two fully-connected layers (FC).

Supplementary Table 1. Signal variables of MLP in the bicycle experiment.

Name	I/O	Description	Domain	unit
φ_1	I	Target angle of inclination	[-12, 12]	deg
φ_2	I	Adjusted angle of inclination	[-15, 15]	deg
ω	I	Adjusted angular velocity	[-25, 25]	deg/s
v	I	Velocity of bicycle	[0, 2.5]	m/s
k_v	I	Voltage coefficient	[0.8, 1.6]	-
φ_3	O	Target rotation angle	[-60, 60]	deg

Supplementary Table 2. The definition of states and relative triggers and modules.

State	Definition	Triggers	Relative modules
S0	Initial state	T6: Straight command<br>T5: S2 finished	Motion
S1	Target tracking	T0: Follow command<br>T8: Finding person	Visual, Motion
S2	Speed change	T1: Speed command	Auditory, Motion
S3	Turning	T2: Turning command	Auditory, Motion
S4	Obstacle avoiding	T3: Finding obstacle	Visual, Motion
S5	Target seeking	T4: No person in view<br>T7: Turn ready	Visual, Motion

Supplementary Methods

Notation of CNN The output of CNN contains only one grid cell and are encoded as a (4+1)×2 tensor denoting the central coordinates (x, y), the width (w) and height **h** and the confidence **c** for both human and obstacle. To stabilize training and avoid numerical problems, the output values w and h are the scaling factor for two anchors, while the central coordinates (x, y) are the bias ratio adjusted by sigmoid nonlinearity:

$$[x, y] = sigmoid(O_x,\ \ O_y) \tag{1}$$

$$[w, h] = \exp(O_w,\ \ O_h) * anchor \tag{2}$$

$$c = sigmoid(O_c) \tag{3}$$

where the default anchor (width, height) for human and obstacle are the average values in the training dataset. The network is jointly trained with weighted sum of different losses:

$$L_{xy} = \sum_{obj} \mathbb{C}_{obj}[(x - \hat{x})^2 + (y - \hat{y})^2] \tag{4}$$

$$L_{wh} = \sum_{obj} \mathbb{C}_{obj}[(\sqrt{w} - \sqrt{\hat{w}})^2 + (\sqrt{h} - \sqrt{\hat{h}})^2] \tag{5}$$

$$L_c = \sum_{obj} \mathbb{C}_{obj}(c - \hat{c})^2 \tag{6}$$

$$L = \lambda_{xy} L_{xy} + \lambda_{wh} L_{wh} + \lambda_c L_c \tag{7}$$

where $\mathbb{C}_{obj}$ denotes if object appears and $obj \in \{human, obstacle\}$, $\hat{\bullet}$ denotes labels and λ denotes weight of the loss. We find that decreasing λ_c helps training, and the tracking task mostly focus on the central position, so we set $\lambda_{xy}=1, \lambda_{wh}=0.5, \lambda_c=0.1$. After Optimizing the architecture of CNN, the final results of (L_{xy}, L_{wh}, L_c) is (26, 18, 23)$\times 10^{-5}$.

Auditory Data pre-processing The voice recognition is a six-instruction task (including 'left', 'right', 'straight', 'speed up', 'slow down', 'follow me' instructions). To obtain enough training data, we collect these commands announcing by several people for several times in the playground, a real-world scenario. After elaborate collections (e.g. remove some data with too high signal-to-noise rate), we retain a total of 690 instructions as the

dataset. For training and testing, we randomly partition them into test and training subsets by a ratio of 5:1.

During data pre-processing, an end-point detection method based on short-term energy was used to detect and abstract the useful speaker information. After getting segmentations from raw audio stream, we take MFCC, a well-used speech signal processing method inspired by the processing mechanism of the cochlear, to get different frequency features. Specifically, for each frame of segmented signal, MFCC maps it into different mel frequent coefficients. For each MFCC feature, we normalize a feature variable x to the range [0, 1], yielding

$$x = \frac{x - x_{\min}}{x_{\max} - x_{\min}} \tag{8}$$

Then, we adopt the Gaussian population coding strategy to encode the preprocessed data. When receiving the stimulus, the corresponding Gaussian neuron *j* calculates the distance between the input values x_j and receptive center u_{ij} u_{ij}, and fires a spike following the probability

$$p_{ij} = \frac{1}{\sqrt{2\pi\sigma_{ij}}} e^{-\frac{(x_i - u_{ij})^2}{2\sigma_{ij}}} \tag{9}$$

For each MFCC features, we use a Gaussian population consisting of 10 neurons with different respective fields to encode it into spike trains.

Sub-parts of motion module The motion control module needs to generate the steering angle to help the bicycle maintain balance and follow desired trajectories, which mainly contains two steps: generating sequential action signals and integrating all sensor signals to target rotation angle.

In step one, the action instructions can be set manually or generated by high-level module. We design three motion functions that contains certain motion patterns to generate sequences of action from a decision:

(**1**) **Turning command:** The turning core transform one spike turning command to a smooth angle sequence. The turning curve we applied can be formulate as:

$$y(t) = kA\sin\frac{\pi t}{N}, t \in [1, 2, 3, \cdots, N] \quad (10)$$

where k is a coefficient, A is the sign of the signal which represents the direction (minus indicates turn left while positive indicates right), and t denotes the time step. For single turning command, the $t_{\max}$can be set less than N/2 and when $t > t_{\max}$, y will maintain as $y(t_{\max})$. For 'force turn' instruction, N is 100 and $t_{\max}$is equal to N, which means this core produces a sequential value in the following 100 time-steps, indicating a specialized turning pattern with reset.

(**2**) **Tracking function:** The tracking core continuously receives the detection results from CNN network. The tracking error is to smoothed by an exponential moving average:

$$e_{t+1} = \alpha e_t + (1-\alpha)\cdot(x_{t+1} - 0.5) \quad (11)$$

where α is a coefficient and set to 0.8, x is the tracking result x and ranges from [0,1]. Then the averaged error and the result of turning are summed as the final target angle of inclination φ_1.

(**3**) **Speed Adjust:** This core receives two types of spike signals indicating speed up and slow down instructions, respectively. The speed of the bicycle is controlled by a voltage coefficient. Then the core produces piecewise function of the voltage coefficient. To smooth the speed changes and stabilize the motions, any changes are performed linearly with 200 times steps, which gives that:

$$k_v^{t+n} = clip(k_v^t + clip(\frac{n}{200}, 0, 1)\cdot\delta, 0.8, 1.6) \quad (12)$$

where δ notes the value in a single adjustment. We set δ to 0.4, then the bicycle has 3 types of speed.
